# Intestinal Diffuse Large B-Cell Lymphoma in a Patient with Systemic Lupus Erythematosus

**DOI:** 10.1155/2020/7947540

**Published:** 2020-04-09

**Authors:** Masaya Iwamuro, Takahide Takahashi, Yoko Ota, Takehiro Tanaka, Noboru Asada, Shuya Yano, Mayu Uka, Rei Nakamura, Yuki Baba, Seiji Kawano, Yoshiro Kawahara, Hiroyuki Okada

**Affiliations:** ^1^Department of Gastroenterology and Hepatology, Okayama University Graduate School of Medicine, Dentistry, and Pharmaceutical Sciences, Okayama 700-8558, Japan; ^2^Division of Medical Support, Okayama University Hospital, Okayama 700-8558, Japan; ^3^Department of Pathology & Experimental Medicine, Okayama University Graduate School of Medicine, Dentistry, and Pharmaceutical Sciences, Okayama 700-8558, Japan; ^4^Department of Pathology, Okayama University Graduate School of Medicine, Dentistry, and Pharmaceutical Sciences, Okayama 700-8558, Japan; ^5^Department of Hematology and Oncology, Okayama University Hospital, Okayama 700-8558, Japan; ^6^Department of Gastroenterological Surgery, Okayama University Graduate School of Medicine, Dentistry, and Pharmaceutical Sciences, Okayama 700-8558, Japan; ^7^Department of Radiology, Okayama University Hospital, Okayama 700-8558, Japan; ^8^Department of Nephrology, Rheumatology, Endocrinology and Metabolism, Okayama University Graduate School of Medicine, Dentistry, and Pharmaceutical Sciences, Okayama 700-8558, Japan; ^9^Department of Practical Gastrointestinal Endoscopy, Okayama University Hospital, Okayama 700-8558, Japan

## Abstract

A 44-year-old Japanese woman with systemic lupus erythematosus (SLE) presented to our hospital with abdominal pain. Radiological and endoscopic examinations led to the diagnosis of diffuse large B-cell lymphoma of the jejunum, which was subsequently resected. Patients with SLE reportedly have an increased risk of non-Hodgkin lymphoma, as demonstrated by our patient. Hence, lymphoma should be considered in the differential diagnosis of neoplastic lesions emerging in SLE patients. In addition, flow cytometry using endoscopically biopsied fragments is useful for the immediate diagnosis of lymphoma, leading to timely and accurate preoperative staging.

## 1. Introduction

Systemic lupus erythematosus (SLE) is a multifactorial autoimmune disease that affects various organs in the body. SLE occasionally affects the gastrointestinal tract, leading to bowel wall thickening, hemorrhage, obstruction, perforation, infarction, or pancreatitis owing to serositis and mesenteric vasculitis [1, 2]. In addition to inflammation, SLE is reportedly linked to an increased risk of malignant diseases such as cancers of the esophagus, stomach, hepatobiliary complex, cervix, vagina/vulva, kidney, bladder, lung, oropharynx, larynx, skin, and thyroid [3–5]. SLE patients have a particularly increased risk of lymphoproliferative disorders. Malignant diseases are one of the major causes of morbidity and mortality in SLE patients, and prompt diagnosis and treatment are keys to their successful management.

We report a case of an SLE patient who presented with postprandial abdominal pain. Radiological, endoscopic, and pathological examinations led to the diagnosis of diffuse large B-cell lymphoma in the jejunum. Of note, double balloon enteroscopy and flow cytometry analysis using endoscopically biopsied fragments were useful for the immediate diagnosis of lymphoma, leading to timely and accurate preoperative staging.

## 2. Case Presentation

A 44-year-old Japanese woman presented with postprandial abdominal pain since 2 months. She had been diagnosed with SLE at the age of 37 years. The patient also had lupus nephritis, Basedow's disease, steroid diabetes, idiopathic thrombocytopenic purpura, and hypertension, for which she had been taking tacrolimus, azathioprine, hydroxychloroquine, prednisolone (10 mg/day), nifedipine, eplerenone, hydralazine, lansoprazole, propylthiouracil, alfacalcidol, and sodium ferrous citrate. She was a social drinker and an ex-smoker who smoked 30 cigarettes/day for 10 years. On examination, her body temperature was 36.7°C, blood pressure was 123/80 mmHg, and pulse rate was 103 bpm. Her height was 148.5 cm and weight was 42.4 kg. Physical examination revealed conjunctival anemia, exophthalmos, enlarged thyroid, and distended abdomen with hyperactive bowel sounds, but there was no palpable mass or tenderness in her abdomen. Laboratory tests showed decreased values for hemoglobin concentration (9.0 g/dL) and hematocrit (28.3%). The C-reactive protein (4.11 mg/dL), erythrocyte sedimentation rate (104 mm/h), and soluble interleukin-2 receptor (779 U/mL) levels were elevated. The white blood cells, platelets, lactate dehydrogenase, creatine phosphokinase, anti-double stranded DNA IgG antibody, complements, carcinoembryonic antigen, and carbohydrate antigen 19–9 were within the normal range. Urinalysis revealed proteinuria, leukocytes (20–29 cells/high power field), and tubular epithelium. Hematuria was absent.

**Figure 1:**
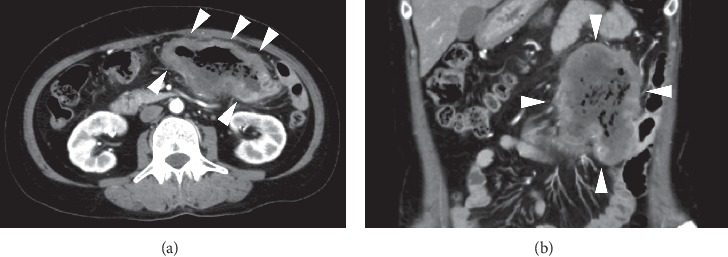
Computed tomography scanning images. Whole circumferential thickening of the jejunum is shown (arrows) (a), (b).

**Figure 2:**
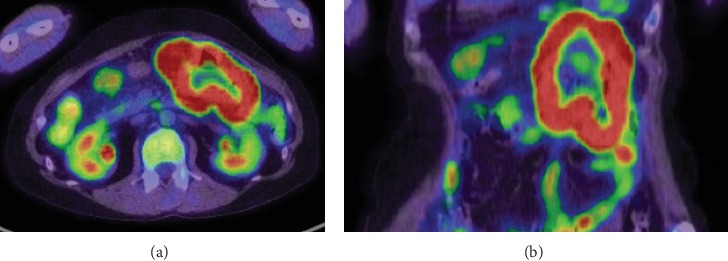
Positron emission tomography scanning images. Tracer uptake is observed in the thickened intestinal wall.

**Figure 3:**
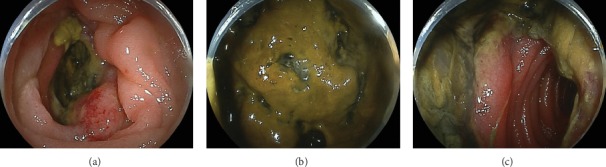
Double balloon enteroscopy images. A circumferential ulcer with necrotic debris in the jejunum is shown.

**Figure 4:**
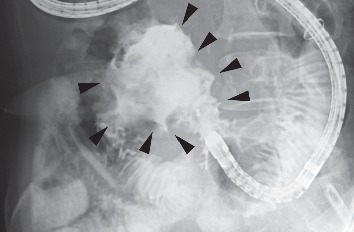
A contrast study image during enteroscopy. An irregularly dilated jejunal lumen is observed (arrows).

**Figure 5:**
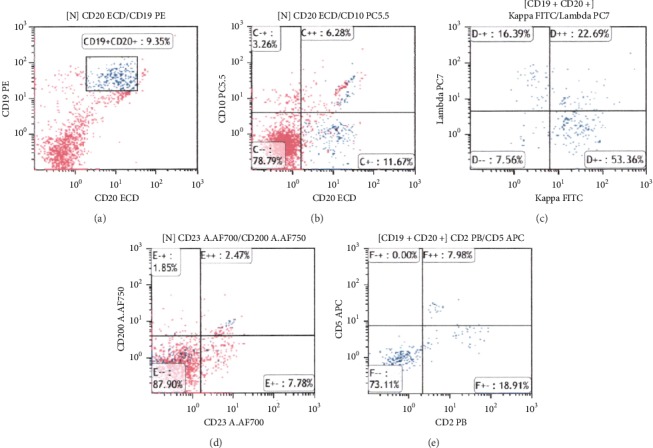
Flow cytometry results. We performed flow cytometry using 2 endoscopically biopsied fragments. The κ/λ ratio of CD19+/CD20+ cells is 3.3 (53.36/16.39), indicating positive light chain restriction.

**Figure 6:**
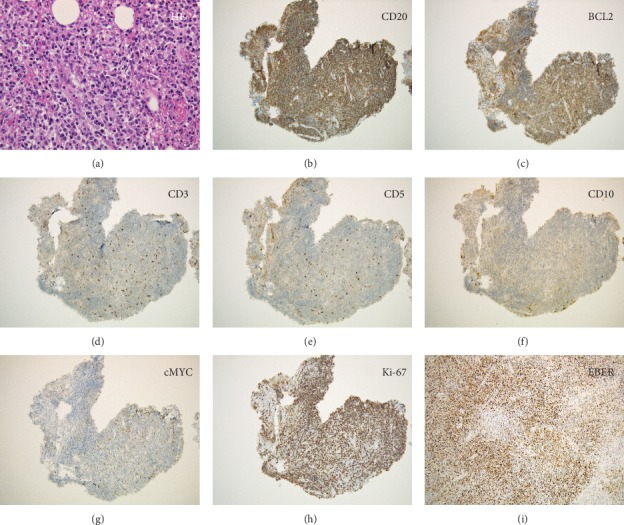
Pathological images. Infiltration of atypical, large lymphoid cells is seen in the endoscopic biopsy specimens (a): Hematoxylin-eosin stain. Lymphoma cells are positive for CD20 (b) and BCL2 (c), while they are negative for CD3 (d), CD5 (e), CD10 (f), and cMYC (g). The cells are diffusely positive for Ki-67 (h). The results of in situ hybridization for Epstein–Barr virus-encoded small RNA-1 (EBER) are also positive (i).

Computed tomography scanning showed whole circumferential thickening of the jejunum with aneurysmal dilatation, which is a typical feature of malignant lymphomas of the intestine (Figure 1). On positron emission tomography scanning, tracer uptake was observed in the thickened intestinal wall (Figure 2). Double balloon enteroscopy revealed circumferential ulcer and necrotic debris in the jejunum (Figure 3). A contrast study during enteroscopy showed dilated jejunal lumen (Figure 4). Based on the morphology of the jejunal lesion, we suspected small intestinal lymphoma rather than cancer. Thus, we performed flow cytometry analysis with 2 endoscopically biopsied fragments, as described previously (Figure 5) [6]. The *κ*/*λ* ratio of CD19 + CD20+ cells was 3.3 (53.36/16.39), indicating monoclonal proliferation of the B cells producing the *κ* light chain. The isolated cells were negative for CD5 and CD10 on flow cytometry analysis. Bone marrow biopsy and cytology revealed no lymphoma cell invasion. Biopsy of the jejunal lesion showed infiltration of atypical, large lymphoid cells that were positive for CD20 and BCL2 and negative for CD3, CD5, CD10, and cMYC (Figure 6). The cells were diffusely positive for Ki-67. The results of in situ hybridization for Epstein–Barr virus-encoded small RNA-1 were also positive. The jejunal tumor, which had invaded the transverse colon, was surgically resected. Lymphadenopathies of the mesentery were observed intraoperatively. Consequently, a diagnosis of diffuse large B-cell lymphoma in the jejunum at stage II_1E_ (large intestine) was made. Chemotherapy with cyclophosphamide, adriamycin, vincristine, and prednisone plus rituximab was administered postoperatively.

## 3. Discussion

Patients with SLE and other rheumatic diseases have an increased risk of malignant diseases, of which lymphoma is the most common neoplasm [7–9]. A nationwide population-based study in Taiwan revealed that among the 16,417 patients with SLE, 512 developed cancers (3.1%), including 34 with non-Hodgkin lymphoma (0.2%) [10]. The standardized incidence ratio was the highest for non-Hodgkin lymphoma (4.2, 95% CI: 2.9–5.9) on site-specific cancer risk analysis. A meta-analysis also showed that the pooled standardized incidence ratio for non-Hodgkin lymphoma in patients with SLE was 4.93 (95% CI: 3.81–6.36). SLE is considered to be correlated with an increased risk of non-Hodgkin lymphoma, and the risk is estimated to be 4–7 times greater than that in the general population [7]. In addition, diffuse large B-cell lymphoma is the most common subtype of lymphoma, accounting for 37–62% of all lymphomas in SLE patients [11–13]. Therefore, diffuse large B-cell lymphoma should be considered a probable diagnosis when tumorous lesions are found in patients with SLE.

Several mechanisms can be hypothesized in the pathogenesis of non-Hodgkin lymphoma in SLE patients. First, chronic inflammation may lead to hyperactivity and proliferation of B lymphocytes, resulting in autonomous, monoclonal expansion [14]. Second, dysregulation of the immune system may cause a defective clearance of apoptotic cells [7, 15]. Third, it has been reported that immunosuppressants such as cyclophosphamide are partly responsible for the increased incidence of lymphoma in patients with SLE [16]. Fourth, the Epstein–Barr virus infection may be a common trigger factor for SLE and non-Hodgkin lymphoma [10]. Previous studies have revealed that patients with SLE have an elevated viral load, increased Epstein–Barr virus mRNA expression, elevated levels of Epstein–Barr virus-directed antibodies, and decreased cell-mediated immunity against the virus when compared to healthy controls [17]. The Epstein–Barr virus infection results in the production of the viral protein “Epstein–Barr virus nuclear antigen-1” (EBNA-1). Antibodies against EBNA-1 cross react with the autoantigens found in SLE, including Ro, Sm B/B′, and Sm D1 [18]. Thus, molecular mimicry may play a crucial role in the pathogenesis of SLE. Simultaneously, the Epstein–Barr virus can also promote lymphomagenesis [19]. We believed that the Epstein–Barr virus played a pivotal role in the development of diffuse large B-cell lymphoma in our patient because the in situ hybridization for Epstein–Barr virus-encoded small RNA-1 was positive.

It was also noteworthy that flow cytometry analysis using 2 endoscopically biopsied fragments was useful for the prompt diagnosis of lymphoma in our patient. Since B-cell lymphomas typically arise from a massive expansion of a B-cell clone, neoplastic cells express only 1 class of immunoglobulin light chain, which is either *κ* or *λ* chain. Thus, immunoglobulin light-chain expression can be used as a surrogate marker for clonality. However, flow cytometry has not been widely used in routine clinical practice for the diagnosis of gastrointestinal lymphoma, mainly because an appropriate protocol is absent. We have recently established lymphocyte isolation techniques and revealed that light chain expression analysis could be performed with 2 endoscopic biopsy fragments obtained from 10 patients (6). Although pathological analysis is still a standard for the diagnosis of lymphoma subtype, flow cytometry analysis has the advantage of delivering faster results; while several hours are required to obtain the results of flow cytometric analysis, and histological analysis with immunostaining generally takes days to weeks. Based on the flow cytometry results of our patient, we consulted with hematologists on the following day and performed bone marrow aspiration and biopsy 2 days after the endoscopy, resulting in timely preoperative diagnosis.

In conclusion, we encountered an SLE patient with diffuse large B-cell lymphoma in the jejunum. This case reinforces that lymphoma should be considered in the differential diagnosis of neoplastic lesions emerging in SLE patients. Moreover, flow cytometry analysis is useful in making a timely diagnosis of gastrointestinal lymphoma.
